# The progeroid gene BubR1 regulates axon myelination and motor function

**DOI:** 10.18632/aging.101032

**Published:** 2016-09-12

**Authors:** Chan-Il Choi, Ki Hyun Yoo, Syed Mohammed Qasim Hussaini, Byeong Tak Jeon, John Welby, Haiyun Gan, Isobel A. Scarisbrick, Zhiguo Zhang, Darren J. Baker, Jan M. van Deursen, Moses Rodriguez, Mi-Hyeon Jang

**Affiliations:** ^1^ Department of Neurologic Surgery, Mayo Clinic College of Medicine, Rochester, MN 55905, USA; ^2^ Department of Biochemistry and Molecular Biology, Mayo Clinic College of Medicine, Rochester, MN 55905, USA; ^3^ Department of Physical Medicine and Rehabilitation, Rehabilitation Medicine Research Center, Mayo Clinic College of Medicine, Rochester, MN 55905, USA; ^4^ Department of Pediatric and Adolescent Medicine, Mayo Clinic College of Medicine, Rochester, MN 55905, USA; ^5^ Departments of Neurology and Immunology, Mayo Clinic College of Medicine, Rochester, MN 55905, USA

**Keywords:** BubR1, corpus callosum, spinal cord, oligodendrocytes, myelination, motor function

## Abstract

Myelination, the process by which oligodendrocytes form the myelin sheath around axons, is key to axonal signal transduction and related motor function in the central nervous system (CNS). Aging is characterized by degenerative changes in the myelin sheath, although the molecular underpinnings of normal and aberrant myelination remain incompletely understood. Here we report that axon myelination and related motor function are dependent on BubR1, a mitotic checkpoint protein that has been linked to progeroid phenotypes when expressed at low levels and healthy lifespan when overabundant. We found that oligodendrocyte progenitor cell proliferation and oligodendrocyte density is markedly reduced in mutant mice with low amounts of BubR1 (*BubR1*^H/H^ mice), causing axonal hypomyelination in both brain and spinal cord. Expression of essential myelin-related genes such as MBP and PLP1 was significantly reduced in these tissues. Consistent with defective myelination, *BubR1*^H/H^ mice exhibited various motor deficits, including impaired motor strength, coordination, and balance, irregular gait patterns and reduced locomotor activity. Collectively, these data suggest that BubR1 is a key determinant of oligodendrocyte production and function and provide a molecular entry point to understand age-related degenerative changes in axon myelination.

## INTRODUCTION

Aging limits brain plasticity and thus impairs functional integrity of the brain. Myelin, formed by oligodendro-cytes in the central nervous system (CNS), insulates axons and is essential for rapid and efficient transmission of action potentials [1, 2]. Decline in efficiency of myelination-remyelination is associated with increasing age and leads to impaired signal conduction in affected nerves, ultimately causing deficits in movement, motor coordination, balance, as well as cognition [3-5]. However, the molecular mechanisms mediating age-related impairments in oligodendrocyte development and myelination are incompletely understood. BubR1 is a core component of the spindle assembly checkpoint that times sister chromosome separation at anaphase onset with attachment of all chromosomes to the biplor spindle to ensure accurate chromosome segregation over the two daughter cells. As a multi-functional mitotic regulator, BubR1 is implicated in the proper timing of mitosis through APC/C^Cdc20^ signaling, kinetochore-microtubule attachment, and repair of merotelic and synthelic attachment errors through its role in the “attachment error correction machinery” [6, 7]. BubR1 is unique among mitotic regulators in that it is implicated in the development of aging-like pathologies [8, 9]. In wild-type (WT) mice, expression of BubR1 decreases with natural aging in multiple tissues including the testis and ovary. Mice harboring *BubR1* hypomorphic alleles that produce low amounts of BubR1 develop several early onset age-related pathological features, including a short lifespan, lipodystrophy, cataracts, sarcopenia, and cancer [8]. Mutations in the human *BUB1B* gene (encoding BUBR1 protein) are associated with mosaic variegated aneuploidy (MVA) syndrome [10], a childhood syndrome characterized by reduced levels of BubR1 expression and various progeroid features including a short lifespan, short stature, and facial dysmprphisms, cataracts, and cancer predisposition [11, 12]. Interestin-gly, children with MVA syndrome also display microcephaly, developmental delays, CNS abnormalities, and defects in ciliogenesis [10, 13]. Taken together, these clinical and preclinical studies raise the possibility that BubR1 is involved in aspects of brain development, age-related pathologies of the CNS, or both.

While little is known about the neurobiological function of BubR1, demyelinating lesions of patients with multiple sclerosis are characterized by low *BubR1* transcript levels [14], suggesting a potential link between BubR1 insufficiency and myelin-related pathology. Consistent with this notion, BubR1 levels are known to be relatively high in oligodendrocyte progenitor cells (OPCs) [15], and BubR1 binding partners such as Sirt2 and HDAC1 and HDAC2 have been implicated in myelination through oligodendrocyte development [16-19]. This, together with the notion that myelination is critical for both brain development and myelin pathology associated with age- and other neurodegenerative disorders [20-23], led us to explore whether and how BubR1 might contribute to axon myelination using BubR1 hypomorphic (hereafter *BubR1*^H/H^) mice. Here we demonstrate that BubR1 insufficiency impairs oligodendrocyte proliferation and production, leading to defects in myelination and motor-related function. These findings suggest an important role for BubR1 in appropriate oligodendrocyte generation, myelination and motor function.

## RESULTS

### BubR1 insufficiency leads to morphological abnormalities in the postnatal brain

**Figure 1:**
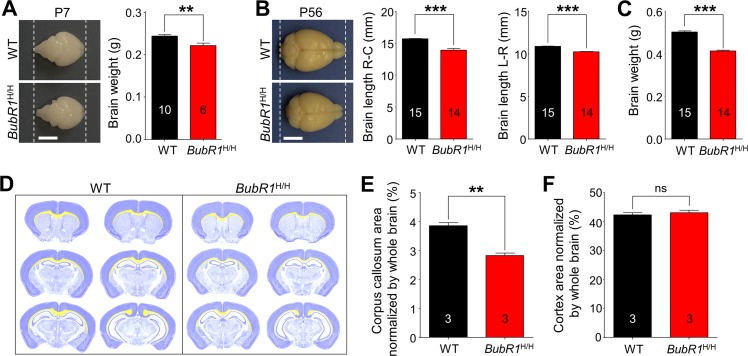
Morphological characterization of BubR1 insufficient mice (**A-C**) Reduced size of *BubR1*^H/H^ mouse brain. (**A**) Representative photographs of WT and *BubR1*^H/H^ mice at postnatal day 7 (P7) and quantification of brain weight. Scale bar: 0.5 cm. (**B**) Representative photographs of WT and *BubR1*^H/H^ mice at postnatal day 56 (P56; 8-week-old). Scale bar: 0.5 cm. *BubR1*^H/H^ exhibit a significantly reduced brain size including rostrocaudal (R-C) length, and width (L-R) (**B**) and weight (**C**). (**D-F**) Deficits in corpus callosum formation in BubR1 insufficient mice. (**D**) Representative images of quantification of cortex and corpus callosum area relative to total section area using cresyl violet stained sections. The cortex is highlighted in dark blue, the corpus callosum is highlighted in yellow. Measurements of the corpus callosum, and cortex area. Arre of the corpus callosum in BubR1 insufficient mouse is significantly different from WT mice (**E**), while cortical area is not (**F**). All values represent mean ± SEM (ns = non-significant, **P < 0.01, ***P < 0.001, student's t-test). Number associated with bar graphs indicates number of animals examined.

To investigate the *in vivo* function of BubR1, we first compared gross morphology of *BubR1*^H/H^ mice. We find that *BubR1*^H/H^ mice are viable and born with no gross alterations [8], but became distinguishable from their WT littermates by their reduced body size and weight (Supp. Fig. 1A). In addition, consistent with a previous report in other tissues including testis and ovary [8], BubR1 mRNA expression is significantly reduced in the hippocampus, spinal cord, and cerebellum of 8-week-old *BubR1*^H/H^ mice relative to their WT littermates (Supp. Fig. 1B). We then compared brain morphology between *BubR1*^H/H^ and WT littermates. While *BubR1*^H/H^ mice show normal brain morphology at birth, thereafter they become morphologically smaller compared to their WT littermates beginning at postnatal day 7 (Fig. 1A), an effect that persists into adulthood as observed by reduced brain size (Fig. 1B) and weight (Fig. 1C). These results indicate *BubR1*^H/H^ mice exhibit normal brain size at birth, but undergo abnormal postnatal development resulting in a reduced brain size.

To determine which brain areas are structurally vulnerable to BubR1 insufficiency, we analyzed coronal sections stained with cresyl violet at 8 weeks of age (Fig. 1D). We show that the area comprising the corpus callosum (highlighted in yellow) is reduced in all coronal planes in *BubR1*^H/H^ mice (corpus callosum area normalized by whole brain; WT: 3.856 ± 0.101%; *BubR1*^H/H^: 2.830 ± 0.079%, *P* = 0.0013) (Fig. 1E), while no difference in cortical area is observed (cortex area normalized by whole brain; WT: 42.32 ± 0.73%; *BubR1*^H/H^: 43.10 ± 0.84%, *P* = 0.5243) (Fig. 1F), suggesting possible postnatal white matter deficits containing corpus callosum. Because one of the major cell types in the white matter is oligodendrocytes, and previous evidence indicates BubR1 is relatively enriched in OPCs compared to other cell types [15], we hypothesized that BubR1 plays a major role in regulating oligodendrocyte development and subsequent myelination.

### OPC proliferation is impaired in the postnatal corpus callosum and spinal cord of BubR1^H/H^ mice

During oligodendrocyte development, OPCs proliferate and differentiate to become mature oligodendrocytes, generating myelin and providing essential trophic support for axons [24]. While BubR1 is known as a cell cycle regulator in proliferating cells [25], whether BubR1 also regulates OPC proliferation is not known.

**Figure 2:**
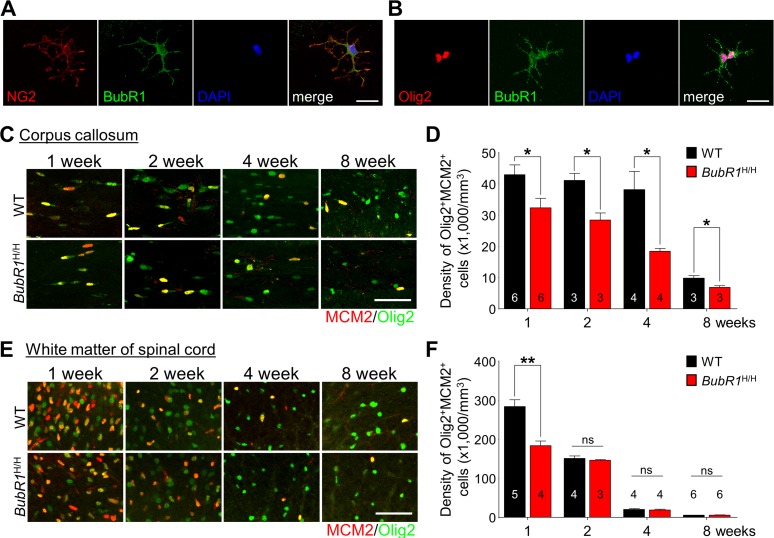
BubR1 insufficiency impairs OPCs proliferation during oligodendrocyte development in the CNS (**A-B**) BubR1 expression in isolated primary oligodendrocyte progenitor cells. Representative image of NG2^+^ cell (a marker for oligodendrocyte progenitor cells) (**A**). Representative image of Olig2^+^ cell (a marker for oligodendrocyte lineage cells) (**B**). Scale bar: 25 μm for (**A**) and (**B**). (**C-D**) The number of proliferating oligodendrocyte lineage cells in the corpus callosum. (**C**) Representative images of MCM2^+^Olig2^+^ cells within the corpus callosum at 1, 2, 4, and 8 weeks old. (**D**) Quantification of MCM2^+^Olig2^+^ cell number in the corpus callosum. Scale bar: 50 μm. (**E,F**) The number of proliferating oligodendrocyte lineage cells in the white matter of spinal cord. (**E**) Representative images of MCM2^+^Olig2^+^ cells in the white matter of spinal cord at 1, 2, 4, and 8 weeks old. Scale bar: 50 μm. (**F**) Quantification of proliferating OPCs (Olig2^+^MCM2^+^) in the white matter of spinal cord. All values represent mean ± SEM (ns: non-significant, *P < 0.05, **P < 0.01, student's t-test). Number associated with bar graphs indicates number of animals examined.

To address this question, we first confirmed BubR1 expression in isolated primary OPCs *in vitro*. Our immunostaining analysis shows BubR1 expression in both NG2^+^ (OPCs marker; Fig. 2A) and Olig2^+^ (oligodendrocyte lineage marker; Fig. 2B) OPCs, suggesting a potential role of BubR1 in OPC pro-liferation and/or differentiation. We next determined whether BubR1 regulates OPCs proliferation *in vivo* by analyzing proliferating OPCs defined by Olig2 and proliferation marker MCM2 [26] at 1, 2, 4, and 8 weeks of age in both corpus callosum (Fig. 2C) and white matter of the spinal cord (Fig. 2E). In the corpus callosum of WT mice, we find that OPC proliferation is highly maintained from 1 until 4 weeks of age after which proliferation declines (Fig. 2D). We also find the number of proliferating OPC (Olig2^+^MCM2^+^ cells) is significantly decreased in *BubR1*^H/H^ mice compared to WT spanning from 1 to 8 weeks of age (Fig. 2D). Independent from MCM2, we also analyzed EdU, a new thymidine analogue [27] with a 2 hour pulsing chase paradigm in 1-week-old WT and *BubR1*^H/H^ mice, and analyzed Olig2^+^EdU^+^ cells to confirm the level of proliferating OPCs (Supp. Fig. 2A). Consistent with decreased Olig2^+^MCM2^+^ cells, we also found a decrease of Olig2^+^EdU^+^ proliferating OPCs in the corpus callosum (Supp. Fig. 2B). Together, these data indicate that BubR1 is required for proper OPC proliferation in the postnatal corpus callosum. Interestingly, analysis of OPC proliferation in the spinal cord shows a distinct timeline of proliferation. As shown in Fig. 2F, OPC proliferation is high at postnatal week 1 and decreases by 50% at 2 weeks of age (1 week: 286400 ± 17819 *vs* 2 weeks: 152217 ± 6491), followed by a near cessation at 4 and 8 weeks in the spinal cord of WT mice. Relative to WT mice, we find that *BubR1*^H/H^ exhibit a significant reduction in the number of proliferating OPCs (Olig2^+^MCM2^+^), whereas such deficits do not extend later to 2-8 weeks of age (Fig. 2F), suggesting early defects in OPC proliferation in the spinal cord of *BubR1*^H/H^ mice. After cell cycle exit, proliferating OPCs undergo differen-tiation into pre-oligodendrocyte lineage cells and mature oligodendrocytes (Supp. Fig. 3A) [1]. To examine whether reduction in OPC proliferation is due to premature progression into oligodendrocytes, we analyzed the percentage of differentiating Olig2^+^MCM2^−^ oligodendrocyte lineage cells in both the corpus callosum and spinal cord of the *BubR1*^H/H^ mice. We found an age-dependent increase in differentiating oligodendrocyte lineage cells in both corpus callosum and spinal cord of WT mice (Supp. Fig. 3B). BubR1 insufficiency does not alter the proportion of differentiating oligodendrocyte lineage cells in the corpus callosum at 1, 2, and 4 weeks of age, and in the spinal cord at 1 and 4 weeks of age. However, differen-tiating Olig2^+^MCM2^−^ oligodendrocyte lineage cells slightly, but significantly, decreased in 8 week-old *BubR1*^H/H^ corpus callosum, and 2- and 8-week old *BubR1*^H/H^ spinal cord. Together, these results suggest that BubR1 insufficiency results in reduced OPCs proliferation and perhaps subtle impairments in OPC differentiation.

### BubR1 insufficiency limits postnatal oligodendrocyte production in the corpus callosum and spinal cord

**Figure 3:**
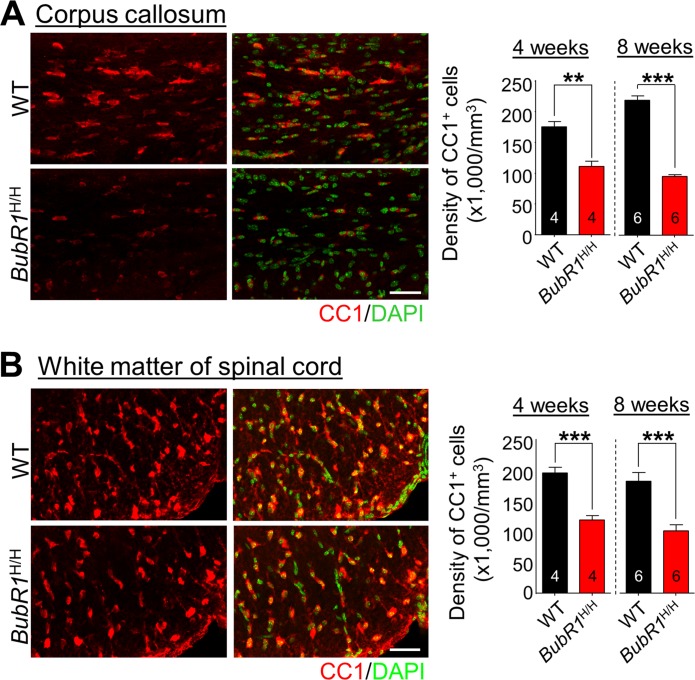
Reductions in mature oligodendrocytes in 4- and 8-week-old BubR1 insufficient mice (**A**) The density of oligodendrocyte lineage cells in the corpus callosum at 4 and 8 weeks of age. Left: Representative images of CC1 staining of 4 week-old WT and *BubR1*^H/H^ mice corpus callosum. Scale bars: 50 μm. Right: Quantification of CC1^+^ cell number in the corpus callosum. (**B**) The density of oligodendrocyte lineage cells in the white matter of spinal cord at 4 and 8 weeks of age. Left: Representative images of CC1 staining of 4 week-old WT and *BubR1*^H/H^ mice spinal cord. Scale bars: 50 μm. Right: Quantification of CC1^+^ cell density in the spinal cord. All values represent mean ± SEM (**P < 0.01, ***P < 0.001, student's t-test). Number associated with bar graphs indicates number of animals examined.

To determine if reductions in OPC proliferation lead to a net change in the overall number of mature oligodendrocytes in *BubR1*^H/H^ mice, we quantified the number of CC1^+^ (a marker for mature oligodendrocytes) cells at later time points (4 and 8 weeks of age). We find a significant decline in the density of CC1^+^ cells in the corpus callosum of *BubR1*^H/H^ mice at both 4 and 8 weeks of age (Fig. 3A). Similarly, early defects in OPC proliferation in the spinal cord of *BubR1*^H/H^ mice also lead to a dramatic reduction in total mature oligodendrocyte production in the white matter of spinal cord at both 4 and 8 weeks of age (Fig. 3B). Taken together, these results demonstrate that BubR1 insufficiency impairs OPC proliferation, resulting in reduced mature oligodendrocyte generation, and suggest an essential function of BubR1 in proper oligodendro-cyte development.

### Axon myelination is impaired in BubR1 insufficient mice

**Figure 4:**
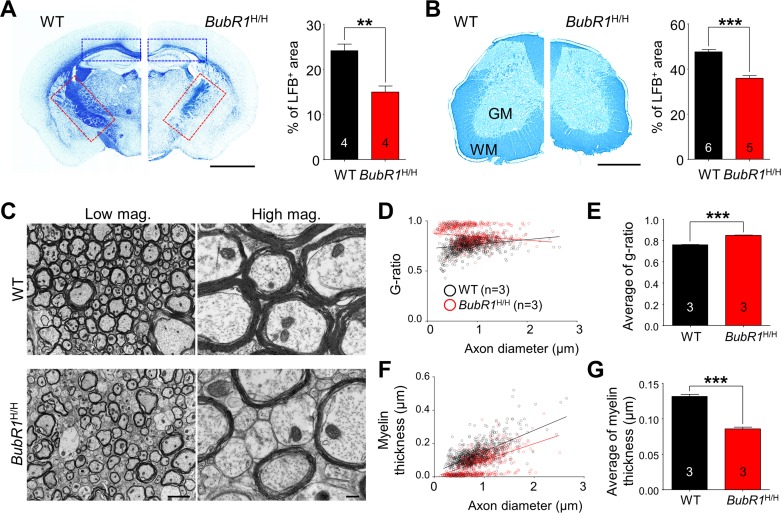
BubR1 insufficiency causes defects in CNS myelination in 8-week-old BubR1 insufficient mice (**A-B**) Luxol fast blue (LFB) staining analysis. (**A**) Sample images of LFB staining in WT and *BubR1*^H/H^ mice (left) and quantification of LFB area in coronal brain sections of 8-week-old mice (right). *BubR1*^H/H^ mice exhibit a profound reduction in myelin density in the corpus callosum (blue dashed box), and internal capsule (red dashed box). Scale bar: 0.2 cm. (**B**) Sample images (left) and quantification of LFB area (right) indicating a profound reduction in myelin density in the spinal cord of *BubR1*^H/H^ mice. Scale bar: 500 μm. WM; white matter, GM; gray matter. (**C-G**) Electron microscopy (EM) imaging analysis of the spinal cord dorsal column white matter of *BubR1*^H/H^ mice and their WT littermates. Hypomyelination is observed in the spinal cord of *BubR1*^H/H^ mice. (**C**) Representative EM images are shown. Scale bars: 2 μm for low magnification, and 0.2 μm for high magnification. (**D,E**) Scatter diagram and quantification of G-ratio. (**F,G**) Scatter diagram and quantification of myelin thickness. All values represent mean ± SEM (**P < 0.01, ***P < 0.001, student's t-test). Number associated with bar graphs indicates number of animals examined.

Our data demonstrate that the density of mature myelin-producing CC1^+^ oligodendrocytes is 56% (corpus callosum; Fig. 3A; *P* < 0.0001) and 44% (spinal cord; Fig. 3B; *P* = 0.0009) less in *BubR1*^H/H^ mice than WT mice at 8 weeks of age, raising the possibility that BubR1 insufficiency affects myelin formation. To explore the possible role of BubR1 in myelination *in vivo*, we performed Luxol fast blue (LFB) staining, a commonly used technique for detecting myelin sheaths [28], on brain and lumbar spinal cord sections of 8-week-old WT and *BubR1*^H/H^ mice. As shown in Figs. 4A and B, *BubR1*^H/H^ mice show a profound reduction in myelin density (hypomyelination) in several brain areas (WT: 24.14 ± 1.45%; *BubR1*^H/H^: 14.94 ± 1.38%, *P* = 0.0036) including corpus callosum (blue dashed box) and internal capsule (red dashed box), as well as white matter of spinal cord (WT: 47.67 ± 0.91%; *BubR1*^H/H^: 35.89 ± 1.19%, *P* < 0.0001) indicating that BubR1 is required for adequate myelination. To determine the structural correlates of myelination defects [29], we also carried out electron microscopy (EM) imaging in the dorsal columns of WT and *BubR1*^H/H^ spinal cord (Fig. 4C). Analysis revealed a significant reduction in axonal myelination in *BubR1*^H/H^ mouse spinal cord as deter-mined by the g-ratio (WT: 0.7595 ± 0.0032; *BubR1*^H/H^: 0.8475 ± 0.0032, *P* < 0.0001, Figs. 4D, and E) and myelin thickness (WT: 0.1318 ± 0.0026μm; *BubR1*^H/H^: 0.0860 ± 0.0023μm, *P* < 0.0001, Figs. 4F, and G). Moreover, EM images show a considerable number of unmyelinated axons in *BubR1*^H/H^ mouse spinal cord (Fig. 4C). Altogether, these data demonstrate that BubR1 insufficiency leads to hypomyelination in the postnatal brain and spinal cord, thus suggesting a critical function of BubR1 in maintaining proper myelination in the CNS.

### Expression of oligodendrocyte- and myelin-related genes is reduced when BubR1 is low

**Figure 5:**
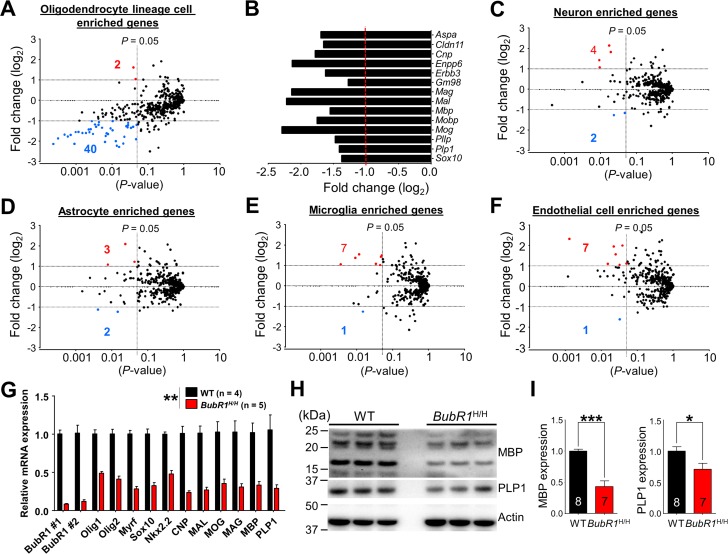
RNA-sequencing analysis reveals reduced expression of oligodendrocyte enriched genes in BubR1 insufficient mice (**A-F**) Scatter plots for visualizing the top 500 genes expressed in each neural cell type, as determined by published RNA-seq data [30], in 8-week-old *BubR1*^H/H^ mice relative to WT. Axes denote fold change (log_2_) by *P*-value. Vertical lines indicate *P*-value of 0.05, horizontal lines fold change (log_2_) of ±1. Red dots and corresponding number indicate up-regulated genes, blue dots and corresponding number indicate down-regulated genes. (**A**) Oligodendrocyte lineage cells enriched genes (2 up, 40 down). (**B**) Graph depicting a representative subset of oligodendrocyte- and myelination-related genes depicted in (**A**). Fold change represents *BubR1*^H/H^ mice with WT as control. (**C**) Neuron enriched genes (4 up, 2 down). (**D**) Astrocyte enriched genes (3 up, 2 down). (**E**) Microglia enriched genes (7 up, 1 down). (**F**) Endothelial cell enriched genes (7 up, 1 down). Number of mice are 3 for each group. (**G**) Validation of mRNA expression of selected genes related to oligodendrocyte development and myelination. mRNA expression of oligodendrocyte development and myelination-related genes were significantly reduced in *BubR1*^H/H^ mice. (**H,I**) Reduced expression of myelin-related proteins in BubR1 insufficient spinal cord. (**H**) Representative Western blot images of MBP and PLP1 in spinal cord lysates from 8-week-old WT and *BubR1*^H/H^ mice. (**I**) Summary of densitometry quantification for MBP (16 and 21 kDa) and PLP1 (30 kDa) protein levels, which was normalized to that of actin for loading controls. All values represent mean ± SEM (*P < 0.05, **P < 0.01, ***P < 0.001, student's t-test). Number associated with bar graphs indicates number of animals examined.

To gain molecular insight into how BubR1 insufficiency affects OPC development and myelina-tion, we performed RNA-seq analysis on isolated hippocampi from 8 week-old adult *BubR1*^H/H^ and WT littermates. We interrogated a public database for 500 genes from the highest enrichment in each neural cell type (neurons, astrocytes, oligodendrocyte lineage cells, microglia, and endothelial cells) [30]. 500 selected genes from each cell-type category are listed in Suppl. Table 1. To visualize any potential effects, in the scatter plot each gene was represented by a dot where the X-coordinate indicates the *p*-value and the Y-coordinate indicates the fold change in *BubR1*^H/H^ mice relative to WT controls. Our RNA-seq analysis indicates that *BubR1*^H/H^ mice exhibit 42 significant oligodendrocyte lineage cell-enriched genes, of which 2 are significantly up-regulated (red dot) and 40 are down-regulated (blue dot) more than 2 fold (Fig. 5A). These down-regulated oligodendrocyte lineage cell-enriched genes include well-known regulatory factors important for oligoden-drocyte differentiation and myelin formation, such as myelin regulatory factor (*gm98/myrf*), SRY (sex determining region Y)-box 10 (*sox10*), and Erb-B2 receptor tyrosine kinase 3 (*erbb3*) for oligodendrocyte differentiation [31-34], and myelin basic protein (*mbp*), myelin-associated oligodendrocyte basic protein (*mobp*), plasmolipin (*pllp*), and proteolipid protein 1 (*plp1*) for myelin formation [35] (Fig. 5B). In contrast, in other neural cell types including neurons (Fig. 5C), astrocytes (Fig. 5D), microglia (Fig. 5E), and endothelial cells (Fig. 5F), we find cell-type specific enriched genes are relatively unchanged by BubR1 insufficiency, with < 8 total affected genes in each. Details on altered gene information in each cell-type category are summarized in Suppl. Table 2. These results were validated using qRT-PCR analysis for selected genes. Consistent with RNA-seq data, qRT-PCR analysis confirmed decreased expression of oligodendrocyte

development and myelination-related genes (Fig. 5G). However, in contrast to 8-week-old *BubR1*^H/H^ mice, we found no alteration of these genes in 1 week-old *BubR1*^H/H^ mice (Supp. Fig. 4). Thus, our gene expression data indicates no change in the major oligodendrocyte- and myelin-related genes at an early postnatal age, but corroborates dramatic reductions at later postnatal ages. In parallel with expression profile in brain region, we also examined the expression level of two major myelin proteins, MBP and PLP1 in the spinal cord by western blotting (Fig. 5H). Similar to results seen in the brain, *BubR1*^H/H^ mice show a dramatically reduced level of MBP (by 57%; WT: 1.00 ± 0.03; *BubR1*^H/H^: 0.43 ± 0.09, *P* < 0.0001) and PLP1 (by 29%; WT: 1.00 ± 0.07; *BubR1*^H/H^: 0.71 ± 0.10, *P* = 0.0273) in the spinal cord (Fig. 5I). Therefore, these results indicate that BubR1 modulates critical oligodendrocyte development- and myelination-associated genes in later postnatal CNS.

### BubR1^H/H^ mice exhibit abnormal motor-related behaviors

**Figure 6:**
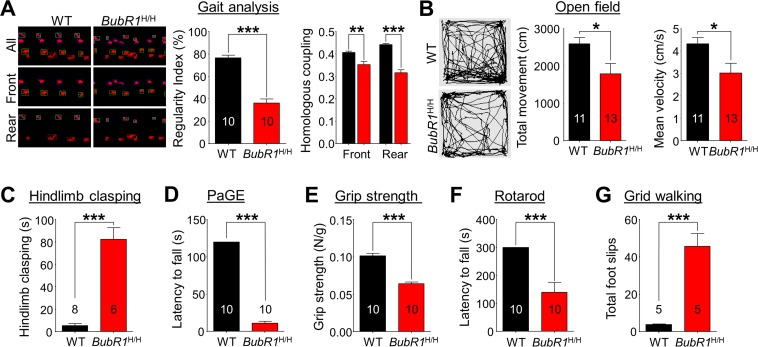
BubR1 insufficient mice exhibit impaired motor function (**A**) Gait analysis. The interlimb coordination was analyzed by the Tread-Scan Gait Analysis System. Left: Examples of severe gait abnormality in *BubR1*^H/H^ mice. Middle: Quantification of symmetry by regularity index (front/rear step). Right: Quantification of homologous coupling (left/right step). (**B**) Locomotion in open field. Left: Representative exploratory activity traces from WT and *BubR1*^H/H^ mice. Middle: Quantification of the total distance travelled. Right: Quantification of the mean velocity. Reduced distance moved in the open field chamber is a measure of reduced spontaneous locomotor activity. (**C**) Hindlimb clasping test. *BubR1*^H/H^ mice exhibit increased hind limb clasping, indicating a motor function abnormality. (**D**) Paw grip endurance (PaGE) test, a measure of balance and endurance, indicates *BubR1*^H/H^ fall sooner than WT littermates. (**E**) Grip strength test. All-paw grip strength is significantly reduced in *BubR1*^H/H^ mice. (**F**) Rotarod test. Performance on a fixed speed rotarod paradigm at 10 rpm was assessed. Quantification of the latency to fall on the rotating platform was significantly reduced in *BubR1*^H/H^ mice. (**G**) Grid walking test. *BubR1*^H/H^ mice exhibit increased total foot slips in the grid walking test. All values represent mean ± SEM (*P < 0.05, **P < 0.01, ***P < 0.001, two-tailed student's t-test). Number associated with bar graphs indicates number of animals examined.

Defects in myelination lead to deficits in motor-related function by impairing signal conduction in affected nerves [36, 37]. Given the observed myelination deficit in *BubR1*^H/H^ mice, we conducted a series of motor-related behavioral tests. To first assess gait and spontaneous locomotor activity of the animals at 8 weeks of age, we performed gait analysis and open field tests, respectively. For gait, the regularity index grades the number of normal step sequence patterns relative to the total number of paw placements, and is used as a measure of the degree of interlimb coordination during the gait cycle (Fig. 6A). We find the regularity index in *BubR1*^H/H^ mice is reduced to 47.4% of WT mice (WT: 76.63 ± 2.38%; *BubR1*^H/H^: 36.29 ± 3.57%, *P* < 0.0001). Homologous coupling defines the phase relationship between two front or rear paws. A coupling value of 0.5 (normal) means that paw contact occurs at 50% of the step cycle of the contralateral limb. We find significant deficits in homologous coupling in *BubR1*^H/H^ mice in both front (WT: 0.4064 ± 0.0066; *BubR1*^H/H^: 0.3525 ± 0.0141, *P* = 0.0028) and rear (WT: 0.4416 ± 0.0051; *BubR1*^H/H^: 0.3157 ± 0.0141, *P* < 0.0001) steps. In addition, *BubR1*^H/H^ mice exhibited decreased spon-taneous locomotor activity as shown by reduction of total movement (WT: 2596 ± 166cm; *BubR1*^H/H^: 1793 ± 270 cm, *P* = 0.0238) in the open field test (Fig. 6B). Our results demonstrate that general movements are compromised in *BubR1*^H/H^ mice.

To further specify *BubR1*^H/H^ mouse motor deficit, we performed motor ability tests including strength and endurance, in addition to overall coordination and balance at 8 weeks of age. Hindlimb clasping is a commonly exhibited phenotype of motor dysfunction associated with Parkinson's disease, Huntington's disease, and cerebellar ataxias [38-40]. Relative to WT mice, *BubR1*^H/H^ mice spend a significantly increased amount of time hindlimb clasping (WT: 5.27 ± 1.83s; *BubR1*^H/H^: 82.36 ± 10.46s, *P* < 0.0001, Fig. 6C). Further testing reveals *BubR1*^H/H^ mice exhibit decreased muscle strength and endurance. In the paw grip endurance (PaGE) test, which requires only balance and grip strength [41], *BubR1*^H/H^ mice exhibit a significant 90% decrease in paw grip endurance compared to their WT littermates (WT: 120.0 ± 0.0s; *BubR1*^H/H^: 11.1 ± 2.5s, *P* < 0.0001, Fig. 6D). In addition, we tested all-paw grip strength using a force meter. We find *BubR1*^H/H^ mice have a reduced lower grip strength (37% less than WT; WT: 0.1015 ± 0.0032N/g; *BubR1*^H/H^: 0.0641 ± 0.0024N/g, *P* < 0.0001, Fig. 6E), suggesting that their decreased latency to fall in the PaGE test may be partly due to an inability to grip.

To identify any deficits in coordination and balance, we performed the rotarod and grid walking tests. *BubR1*^H/H^ mice fall sooner on the rotarod at 10 rpm speed (WT: 300.0 ± 0.0s; *BubR1*^H/H^: 139.2 ± 36.3s, *P* = 0.0003, Fig. 6F). In the grid walking test, *BubR1*^H/H^ mice make more missteps (WT: 3.8 ± 0.2; *BubR1*^H/H^: 45.6 ± 6.9, *P* = 0.0003, Fig. 6G), suggesting they also have poor balance and coordination. Collectively, these data indicate that *BubR1*^H/H^ mice exhibit impaired motor-related function including general movement, as well as balance and coordination.

### Neuronal density in motor-related CNS areas of BubR1^H/H^ mice is unperturbed

**Figure 7:**
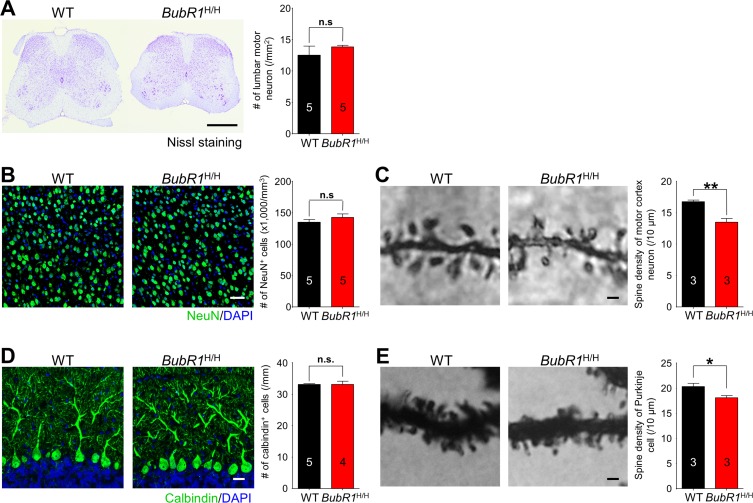
BubR1 insufficiency causes reduction in dendrite spine density of motor neurons and cerebellar purkinje cells (**A**) Motor neuron density in lumbar spinal cord is not significantly different between WT and *BubR1*^H/H^ mice. Left: Representative image of spinal cord with Nissl staining. Right: Quantification of lumbar motor neuron number. Scale bar: 500 μm. (**B**) Neuron density in primary motor cortex is not significantly different between WT and *BubR1*^H/H^ mice. Left: Representative image of NeuN (a neuronal marker) and DAPI staining of motor cortex neurons. Right: Quantification of NeuN^+^ cell density. Scale bar: 50 μm. **(C)** BubR1^H/H^ mouse motor cortex neurons exhibit a reduced dendritic spine density. Left: Representative image of golgi-stained motor cortex neuron dendritic spines. Right: Quantification of dendritic spine density. Scale bar; 1 μm. (**D**) *BubR1*^H/H^ mouse cerebellar Purkinje neuron density is not significantly different from WT mice. Left: Representative image of Calbindin (marker for Purkinje cells) and DAPI staining of Purkinje neurons. Scale bar: 20 μm. Right: Quantification of Calbindin^+^ cell density. **(E)** BubR1^H/H^ mouse cerebellar Purkinje neurons exhibit a reduced dendritic spine density. Left: Representative image of golgi-stained Purkinje neuron dendritic spines. Right: Quantification of dendritic spine density. Scale bar; 1 μm. All values represent mean ± SEM (ns: non-significant, *P < 0.05, **P < 0.01, two-tailed student's t-test). Number associated with bar graphs indicates number of animals examined.

In addition to deficits in myelination, neuronal loss in motor-related areas of the CNS is known to cause motor impairments [42-44]. To explore this possibility, we investigated neuronal density in the major motor-related areas including motor cortex, spinal cord, and cerebellum in 8-week-old *BubR1*^H/H^ mice. Notably, we did not observe significant deficits in neuronal density in the spinal cord (Fig. 7A), motor cortex (Fig. 7B), or cerebellum (Fig. 7D) of adult *BubR1*^H/H^ mice. However, we found a significant reduction in dendritic spine density in the motor neurons (Fig. 7C) and cerebellar purkinje neurons (Fig. 7E) in the *BubR1*^H/H^ mice. Thus, BubR1 insufficiency causes a reduction in dendritic spine density in the motor cortex, and cerebellum, while neuronal density remains unchanged.

## DISCUSSION

CNS myelination relies upon proper differentiation of OPCs into functional oligodendrocytes and the targeting of these newly generated cells with adjacent axons. Impairments in this process occur not only with aging but also in age-related neurodegenerative disorders, CNS injury, and demyelinating disorders including multiple sclerosis, ultimately leading to functional deficits [3, 36, 45, 46]. Thus, a better understanding of the molecular mechanisms that control oligodendrocyte development and myelination is critical towards understanding the fundamental mechanisms of oligodendrocyte biology, and providing insights into potential therapeutic targets for a broad range of myelin-related disorders. Here we uncovered a critical role for the progeroid protein BubR1 in oligodendrocyte development and myelination. We show that BubR1 insufficiency, a hallmark of aging and MVA syndrome, restricts OPC proliferation and oligodendrocyte formation, resulting in postnatal hypomyelination of axons in brain and spinal cord tissue. These impairments are accompanied with reduced expression levels of major oligodendrocyte development- and myelin-related components including gm98, sox10, MBP and PLP1. Functionally, BubR1 insufficiency causes motor related deficits including general movements, balance and coordination in correlation with dendritic spine density in major motor-related areas of the CNS. Collectively, these data identify BubR1 as a key player in oligodendrocyte production and function, and provide a unique starting point for studies into the mechanistic underpinnings of age-related degenerative changes in axon myelination.

Myelination occurs in a stepwise process where OPCs proliferate and mature to become functional myelinating oligodendrocytes. Therefore, precise regulation of OPC proliferation dynamics is key to generating appropriate number of functional oligodendrocytes and maintaining normal myelination [47]. While BubR1 is known as a critical cell cycle regulator in the context of early cortical neural development [48] and tumorigenesis [10], its function in OPC proliferation and their development was not known. Intriguingly, previous studies have identified several components that directly interact with BubR1 in regulating oligodendrocyte development and myelination. For example, HDAC1 and HDAC2, which are shown to interact with BubR1 [16], are specifically implicated in histone deacetylation required for oligodendrocyte differentiation *via* the β-catenin-TCF interaction [17]. In addition, Sirt2 also directly interacts with BubR1 [19], and is implicated as an essential regulator of oligodendrocyte differentiation [18]. It also regulates peripheral myelination through polarity protein Par-3/atypical protein kinase C (aPKC) signaling pathway [49]. Given the critical function of these components that physically interact with BubR1 in regulating oligodendrocyte development and myelination, it is conceivable that BubR1 serves as a critical developmental link between these processes.

We observed that BubR1 insufficiency significantly impairs OPCs proliferation resulting in an overall reduction in the progenitor pool in both the corpus callosum and spinal cord. While we observed this significant reduction in the progenitor pool in the corpus callosum at 1 to 8 weeks of age in *BubR1^H/H^* mice relative to WT, we find that differences in proliferation stabilize in the spinal cord relatively sooner, becoming indistinguishable after just 1 week. These regional differences may exist due to the timeline of development and contributions of respective oligodendrocyte populations in each region. In the spinal cord, a major contribution to the eventual makeup of oligodendrocytes is led by an early population of embryonic OPCs from the ventral ventricular zone [1]. In contrast, OPCs residing in the brain have been demonstrated to come from separate pools and time points across embryonic and postnatal development [50]. Although we observe distinct timelines of OPC proliferation at these two anatomic locations, our data also shows BubR1 insufficiency significantly reduces the OPC proliferation, maturing pre-myelinating and myelinating oligodendrocyte population. However, in addition to critical roles in regulating cell cycle progression [25], BubR1 expression also appears to affect cellular viability. For example, knocking down BubR1 causes massive cell death [51]. Previous studies in mice have also suggested that BubR1 is an essential survival protein, as complete ablation of BubR1 is embryonically lethal due to the extensive apoptosis [52]. We have also observed a slight increase in OPC death in *BubR1^H/H^* mice relative to WT (data not shown). Taken together, although we cannot rule out the possibility that the observed reduction in overall oligodendrocyte population in *BubR1^H/H^* mice may be due to increased OPC death, and perhaps subtle impairments in OPC differentiation (Supp. Info. Fig. 3), our data strongly indicates that BubR1 is required for generating effective numbers of oligodendrocytes.

OPC migration and differentiation into mature oligodendrocytes is a tightly regulated process influenced by the degree of local intrinsic/extrinsic molecular factors and axon-to-oligodendrocyte signaling [24]. While we show BubR1 insufficiency impairs oligodendrocyte generation and causes hypomyelination, how BubR1 mediates this process remains to be established in future studies. In this regard, our RNA-seq analysis reveals that BubR1 insufficiency is associated with a significantly decreased expression of essential genes associated with oligodendrocyte differentiation including *gm98 (myrf)*, *sox10*, and *erbb3* [31-34], and major myelin-related genes including *mbp, mobp, pllp*, and *plp1* [35], while other neural cell-type specific enriched genes in neurons, astrocytes, microglia, and endothelial cells were relatively unchanged by BubR1 insufficiency at later postnatal stages. These results suggest BubR1 may function as a key component modulating a number of oligodendrocyte- and myelin-related genes. Given that these observed genes are highly associated with normal and impaired oligodendrocyte development and myelination [31-34, 53, 54], our results suggest that the presence of BubR1 may be of significant physiological importance to sustain effective levels of essential components associated with oligodendrocyte development as well as myelination, controlling a precise generation of oligodendrocytes, and thus maintaining normal myelination.

We found that impaired oligodendrocyte generation and hypomyelination in *BubR1^H/H^* mice is associated with profound motor deficits. Aging is associated with deficits in balance and motor coordination, leaving older adults prone to serious injury and a reduced quality of life [5, 55]. Given that effective conduction of action potentials depends on appropriate myelination, the age-related loss of myelin function may lead to the conduction delays observed in aging animals and humans [23]. In addition to aging, demyelination and associated motor deficits are observed in patients with multiple sclerosis [56]. Interestingly, a recent microarray analysis shows a reduced level of *BUB1B* in demyelinating lesions of patients with multiple sclerosis [14]. Therefore, it is possible that one of the potential mechanisms whereby BubR1 insufficiency contributes to motor deficits is through regulating myelination. Furthermore, we found BubR1H/H mice exhibit reduced exploratory activity in the open field test, indicating increased anxiety (data not shown). It is likely that defects in myelination and motor function caused by BubR1 insufficiency correlate with increased anxiety, making it consistent with similar observations of increased anxiety associated with aging [57] and multiple sclerosis [58]. Notably, while we found neuronal density in major motor related areas to be unaltered in BubR1 insufficient mice, we observe a reduction in dendritic spine density in motor neurons of the motor cortex and Purkinje neurons of the cerebellum. These data support the notion that BubR1 insufficiency leads to defects in motor function through impaired oligodendrocyte function and myelination, in addition to deficits in neuronal function of major motor related areas, and thus suggest an essential function of BubR1 in maintaining proper myelination and motor ability. The generation and use of *BubR1* conditional knockout and hypomorphic mice to completely or partially knockdown protein expression in a cell type-selective fashion will be a valuable approach to provide further insights into the mechanisms by which BubR1 regulates myelination.

Mutations in the human *BUB1B* gene are linked to MVA syndrome with associated progeroid traits including reduced lifespan, facial dysmorphisms, and short stature [10, 59]. Furthermore, expression levels of BubR1 in WT mice significantly decline with natural aging in multiple tissues [8]. In addition, *BubR1^H/H^* mice develop early onset of premature aging features at 3−6 months of age [8, 9]. However, premature-aging symptoms in BubR1H/H mice occur later compared to our initial observation of hypomyelination. Interesting-ly, Klotho-deficient mice, another proposed premature aging model [60], also exhibit deficiencies in myelination at the early symptomatic period [61]. While here we find BubR1 regulates the proliferation of OPCs, Klotho regulates OPCs maturation through the Akt and ERK signaling pathways [61]. Although different mechanisms may be involved in myelination defects, both models share similar accelerated progeroid symptoms. In addition, Ulrich et al. found a nearly complete absence of mature myelin in a neonatal progeroid syndrome, suggesting a relationship between progeroid features and pathological myelination [62]. Taken together, although supported by less evidence, the relationship between pathological myelination and premature-aging symptoms is of significant interest. Besides its involvement in a suggested aging pathway, the results of our study add an additional role to BubR1, namely, its contribution to myelination and motor function. Importantly, the mechanism underlying these deficits remains an open question for future study.

In summary, we demonstrate a novel function of BubR1 as a critical biological factor that regulates OPC proliferation and oligodendrocyte generation to maintain normal myelination and related motor function. Given that failure in myelination causes impairments in signal conduction in affected nerves, and ultimately loss of motor coordination and balance [3, 36], targeting BubR1 may be of distinct interest towards the development of novel therapies treating myelination-related disorders such as multiple sclerosis, leukodystrophies, and spinal cord injury.

## MATERIALS AND METHODS

### Mice

Mice harboring BubR1 hypomorphic alleles (*BubR1*^H/H^ mice) have previously been described [8]. *BubR1*^H/H^ mice were backcrossed to the C57BL/6 background for over 10 generations. All mice were maintained on a 12 h dark/light cycle throughout the study. Mice had ad libitum access to water and food. All animal protocols and experimental procedures were reviewed and approved by the Mayo Clinic Institutional Animal Care and Use Committee (IACUC).

### Histological analysis

Adult female *BubR1*^H/H^ and their wild-type (WT) littermates at 1, 2, 4, and 8 weeks of age were used for histological analysis.

#### Immunostaining

Coronal brain sections (40 μm in thickness) through the entire brain and spinal cord were prepared in serial order and processed for histological analysis as previously described [63]. For quantification of oligodendrocyte lineage cells, immunostaining was performed with the following primary antibodies: anti-Olig2 (Millipore, Rabbit, AB9610), anti-CC1 (Abcam, Mouse, ab16794), and anti-MCM2 (BD, Mouse, 610701). 4′,6-diamidino-2-phenylindole (DAPI; Sigma) was used for counterstaining. For quantification of motor cortex neuron and cerebellar Purkinje neuron density, immunostaining was performed with the following primary antibodies: anti-NeuN (Millipore, Mouse, MAB377), and anti-Calbindin (Swant, Rabbit, CB-38a). Images were acquired on a Zeiss LSM 780 single-photon confocal system using a multi-track configuration. For counting cells in the corpus callosum, images were acquired to include only the corpus callosum at the midline. In the spinal cord, images were acquired to include only the white matter from serial sections of lumbar spinal cord. Stereological quantification of immunostained cells was carried out using NIH ImageJ [64].

#### Nissl staining

For quantification of spinal motor neurons, lumbar segments of the spinal cord were stained by cresyl violet. All large cells (diameter > 20 μm) containing a distinct nucleus, prominent nucleoli, and at least one thick process in the ventral horn below a lateral line from the central canal were counted.

#### Luxol fast blue staining

Staining for myelin (Luxol fast blue) was performed to evaluate the extent of myelination. Brain sections were defatted, followed by immersion in 0.1% Luxol fast blue solution at 60ºC for 6 h, and 95% ethanol for 5 min. These were then incubated for 1 min in 0.05% lithium carbonate solution, and washed using 70% ethanol and distilled water respectively. After cresyl violet counterstaining, slices were sealed for microscopic observation.

#### Golgi staining

For quantitative analysis of spine density, Golgi staining was performed with Hito Golgi-Cox OptimStain kit (HTKNS1125, HiTO Biote, DE, USA) according to the manufacturer's protocol. 100 μm thick sections were cut with a vibratome (Leica Biosystems, Germany), and visualized under light microscope, using 100X magnifications. At least five neurons were analyzed from three independent *BubR1*^H/H^ mice and their WT littermates.

#### Myelin thickness analysis

The thickness of myelin sheaths was determined by ultrastructural analysis of the spinal cord dorsal column white matter at 8 weeks of age. Mice were perfused with Trump's fixative (4% formaldehyde with 1% glutaraldehyde, pH 7.4) and a 1 mm segment of the lumbar spinal cord was osmicated and embedded in araldite. Myelin sheath thickness was quantified in ultrathin (0.1 mm) sections using a JEM-1400 Transmission Electron Microscope (JEOL USA, Peabody, MA). Images were captured at 8,000X without knowledge of genotype and included four fields across the dorsoventral axis of the dorsal column. G-ratios of at least 250 myelinated axons per mouse were measured using the ImageJ G-ratio plug-in.

### Oligodendrocyte culture

Mouse primary oligodendrocyte progenitor cells (OPCs) were isolated from mixed glial cultures derived from postnatal day 1 mice as previously described [65]. Briefly, primary mixed glial cultures were established from the forebrains of postnatal C57BL/6 mice, and grown in media containing DMEM, 2 mM Glutamax, 1 mM sodium pyruvate, 20 mM HEPES and 10% fetal bovine serum. After 10 days, the flasks were shaken overnight to remove the OPCs [66], and OPCs were seeded on 12 mm glass cover slips coated with poly-L-lysine. At 12 h post-purification cultures, BubR1 expression in OPCs was determined by NG2/BubR1 or Olig2/BubR1 double staining. The following primary antibodies were used: anti-NG2 (Millipore, Rabbit, AB5320), anti-Olig2 (Millipore, Rabbit, AB9610), and anti-BubR1 (BD, Mouse, 612503).

### Validation of MBP and PLP1 expression level in spinal cord

WT or *BubR1*^H/H^ mice spinal cord were homogenized with a Dounce-type glass tissue homogenizer in lysis buffer (10% glycerol and 0.5% NP-40 in phosphate buffered saline (PBS) with protease inhibitor cocktail (Roche)). After centrifuging the crude extract at 4°C for 10 min at 13,000 g, supernatants were collected. Total protein concentration of the supernatant was measured using the bicinchoninic acid (BCA) Kit (Pierce). Whole cell lysates were subjected to SDS-PAGE (4-12% Bis-Tris protein gel) and transferred to nitrocellulose membrane. After blocking with 5% (w/v) skim milk in PBS containing 0.1% Tween 20, the membrane was incubated overnight at 4°C with anti-MBP (cat# MAB386, Millipore) and anti-PLP (cat# ab28486, Abcam) antibody followed by HRP-linked secondary antibody (Cell Signaling Technology). Membranes were then washed and visualized with enhanced chemiluminescence (GE Healthcare Life Science).

### RNA-Sequencing

Total RNA was isolated from the hippocampus of 8-week-old female WT and *BubR1*^H/H^ mice using miRNeasy Mini kit (Qiagen, Valencia, CA). The ovation RNA-seq system v2 kit (NuGEN) was used to prepare RNA-seq libraries according to the manufacturer's instruction. The libraries were sequenced on an Illumina HiSeq 2000 instrument in the Mayo Clinic Center for Individualized Medicine Medical Genomics Facility. Sequence reads from RNA-seq samples were aligned to the mouse genome mm9 and gene annotations from Refseq gene using TopHat v2.05 [67]. In order to compare the gene expression level between WT and *BubR1*^H/H^ mice, the sequencing reads in each gene were counted by BEDTools [68]. The *p*-value was then calculated by DESeq [69].

### Evaluation of motor function

All behavioral tests were performed with female mice at the age of 8 weeks.

#### Hindlimb clasping

Hindlimb clasping is often observed for motor abnormality in a number of mouse models, including certain cerebellar ataxias [40, 70, 71]. To quantify the duration of clasping of the hind limbs, mice were suspen-ded by the tail ∼30 cm above the tabletop for 2 min.

#### PaGE test

Basic grip strength was measured using the paw grip endurance (PaGE) test, which requires only balance and grip strength [41]. Briefly, we measured the time a given animal held on to the inverted lid of cage. Each mouse was given 3 trials with a 10-min rest interval between attempts, and the longest latency was recorded. The cut-off time was 2 min.

#### All-paw grip strength

All-paw grip strength test was assessed using a grip strength meter (Chatillon Ametek Force Measurement, Brooklyn, NY). A grip strength meter calculated the maximum force exerted by a mouse as it was pulled from a grid by the tail. The maximum force output after 3 separate attempts was normalized to the mouse body weight in grams.

#### Rotarod test

Performance of a complex task involving motor coordination, balance, and strength [72, 73] was assessed using a rotarod apparatus (Rotarod Treadmills 47600, Ugo Basile, Varese, Italy) with a 3 cm diameter rod revolving at a constant speed of 10 rpm. Each animal was given three trials, and the longest latency before falling was recorded. An arbitrary cut-off time was set at 300 seconds.

#### Grid walking test

Fine motor coordination was evaluated using the grid walking test [74, 75]. Mice were placed on a wire grid (30 × 35 cm) with 1 cm square holes and allowed to freely explore for 10 min. Performance was recorded with a video camera, and total foot slips of hindlimbs were assessed for 5 min during walking. A footslip was scored either when the animal misplaced its hindlimb to protrude entirely or partially through the grid.

#### Gait analysis

As described previously [76, 77], gait parameters were measured using the Tread-Scan Gait Analysis System (Clever Sys, Reston, VA). Mice were started at 8 cm/s and treadmill speed was adjusted until each mouse maintained a consistent walking speed. The movement of the mouse was then recorded for 60 s at 100 frames per second. TreadScan can produce an assessment of more than 40 gait parameters. Among these parameters, we focused on regularity index, which grades the number of normal step sequence patterns relative to the total number of paw placements, as a measure of the degree of interlimb coordination during the gait cycle.

Spontaneous activity in open field: To monitor spontaneous activity, mice were placed in an open field chamber (40 × 40 cm), and allowed to explore freely for 10 min. Mouse behavior was recorded by a video camera positioned on the ceiling in the center of the testing room. The total movement and mean velocity were analyzed using video-tracking program EthoVision XT 10 (Noldus Information Technology Inc., Leesburg, VA).

### Quantitative RT-PCR (qRT-PCR)

Total RNA was isolated using the RNeasy Mini Kit (QIAGEN). cDNA was generated using SuperScript III Reverse Transcriptase (Invitrogen) according to the manufacturer's protocol, and qRT-PCR was performed using a Bio-Rad CFX Connect real-time PCR detection system (Bio-Rad) with SYBR Green Master Mix (Bio-Rad). Briefly, RNA was initially denatured for 5 min followed by 40 cycles of denaturing at 95°C for 15 sec, and annealing/elongation at 60°C for 1 min.

To confirm BubR1 expression, hippocampal, cerebellar, and spinal cord tissue was obtained from both female WT and *BubR1^H/H^* mice. The following primer sequences were used: β-actin: 5′-TTCTACAATGAGCTGCGTGTG-3′ (forward), 5′-GGGGTGTTGAAGGTCTCAAA-3′ (reverse), BubR1: 5′-CCAGCTGAAGGTTGAGGGAG-3′ (forward), 5′-TGAAGTGTGGACATGACCCG-3′ (reverse).

To validate oligodendrocyte related genes, the corpus callosum tissue was removed from gross coronal sections at approximately Bregma 1 mm and −2 mm. Sagittal cuts were then made through the cingulum, medial to each lateral ventricle, followed by a cut above and below the corpus callosum to remove the majority of cortex and hippocampus [78]. The primer sequences for oligo-dendrocyte related genes are listed in Suppl. Table 3.

### Statistics

All statistical analysis was performed using GraphPad Prism version 6.00 for Windows (GraphPad Software, La Jolla, California). The Student's *t*-test was chosen to determine statistical significance between two groups.

## SUPPLEMENTARY MATERIAL FIGURES AND TABLES


