# Supplementary material for: The progeroid gene BubR1 regulates axon myelination and motor function
Source: Aging (Albany NY). 2016 Sep 12;8(11):2667–84. doi: 10.18632/aging.101032 (PMC5191862; doi:10.18632/aging.101032)
Supplement: Supplementary file 1 [file aging-08-2667-s001.pdf]

## SUPPLEMENTARY MATERIAL

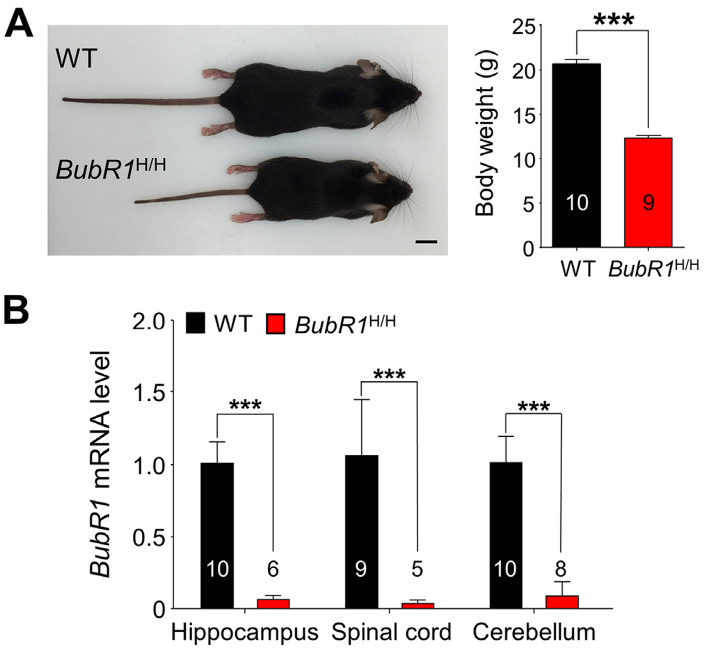


**Supplementary Figure 1. Body size and BubR1 expression level in 8 weeks old BubR1 insufficient mice.** (**A**) *BubR1*$^{H/H}$ mice are significantly smaller than WT mice. Left: Representative image of WT and *BubR1*$^{H/H}$ mice at 8 weeks of age. Right: Reduced body weight and length in *BubR1*$^{H/H}$ mice. Scale bar = 1 cm. (**B**) qRT-PCR experiments indicate reduced *BubR1* mRNA in isolated adult mouse hippocampi, spinal cord, and cerebellum. All values represent mean ± SEM (***$P$ < 0.001, student's t-test). Number associated with bar graphs indicates number of animals examined.

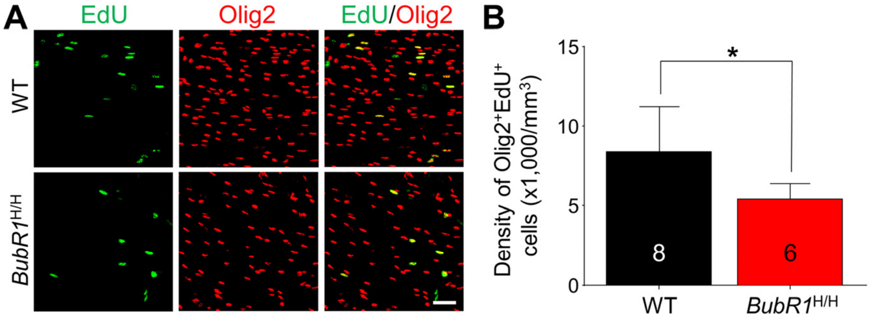


**Supplementary Figure 2. Reductions in proliferating OPCs in the corpus callosum of 1-week-old BubR1 insufficient mice.** (**A**) Representative images of EdU (green) and Olig2 (red) staining of 1 week-old WT and *BubR1*$^{H/H}$ mice corpus callosum. WT and *BubR1*$^{H/H}$ mice were injected with a single dose of EdU (41.2 mg/kg body weight, i.p.) and sacrificed after 2 hours. Scale bars: 100 µm. (**B**) Quantification of EdU$^{+}$Olig2$^{+}$ cell density in the corpus callosum. All values represent mean ± SEM (*$P$ < 0.05, student's t-test). Number associated with bar graphs indicates number of animals examined.

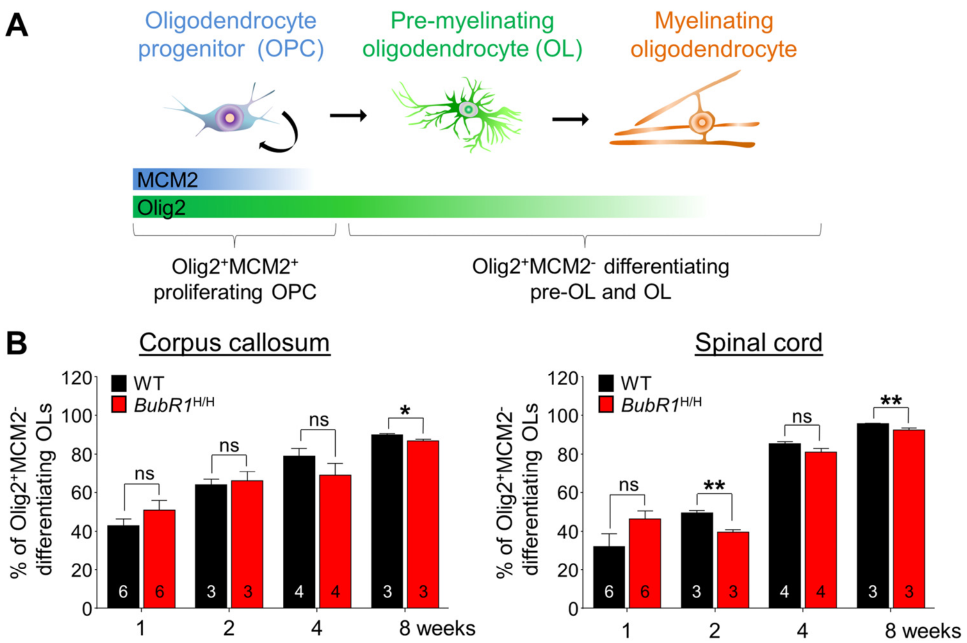


**Supplementary Figure 3. The proportion of proliferating OPCs among total Olig2$^{+}$ cells in BubR1 insufficient mice at different ages.** (**A**) Schematic representation of the proportion of proliferating oligodendrocyte progenitor cells (OPCs) vs. differentiating oligodendrocytes (OLs) among total Olig2$^{+}$ cells. (**B**) Quantification of differentiating pre-OLs and OLs (Olig2$^{+}$MCM2$^{-}$) in the corpus callosum (left) and white matter of spinal cord (right). All values represent mean ± SEM (ns: non-significant, *$P$ < 0.05, **$P$ < 0.01, student's t-test). Number associated with bar graphs indicates number of animals examined.

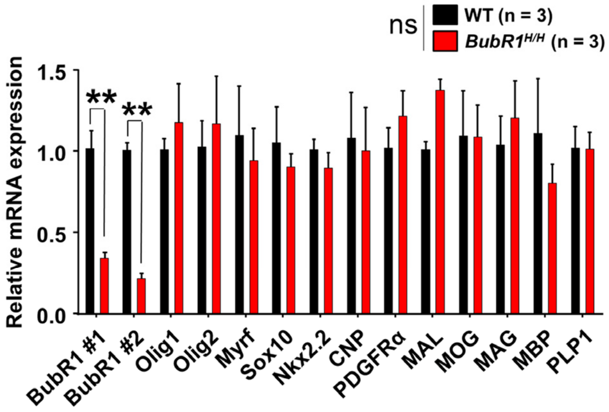


**Supplementary Figure 4. No changes in selected genes related to oligodendrocyte development and myelination in 1-week-old BubR1 insufficient mice.** Quantification of mRNA expression of oligodendrocyte development and myelination-related genes were significantly reduced in *BubR1*$^{H/H}$ mice at 1 week by qRT-PCR. All values represent mean ± SEM (ns: non-significant, **$p$ < 0.01, student's t-test).

**Supplementary Table 1. Summary list of enriched genes in BubR1 insufficient mice by RNA-sequencing analysis.** Please browse the Full text version to see the data of Supplementary Table 1.

**Supplementary Table 2. Summary of significantly enriched genes in BubR1 insufficient mice by RNA-sequencing analysis.**

| | Gene | *p*-value | F.C.($log_2$) |
|---|---|---|---|
| Oligodenrocyte lineage cells | Mog | 0.01582 | -2.29 |
| | Mal | 0.01367 | -2.22 |
| | Mag | 0.00024 | -2.14 |
| | Enpp6 | 0.00091 | -2.13 |
| | Pdlim2 | 0.02058 | -2.12 |
| | Plekhh1 | 0.00644 | -2.04 |
| | Opalin | 0.01285 | -1.99 |
| | Insc | 0.04700 | -1.91 |
| | Bcas1 | 0.00032 | -1.86 |
| | Plxnb3 | 0.02214 | -1.79 |
| | Cnp | 0.00059 | -1.78 |
| | Mobp | 0.00670 | -1.75 |
| | Aspa | 0.00071 | -1.69 |
| | Gpr17 | 0.00048 | -1.68 |
| | Ninj2 | 0.00780 | -1.65 |
| | Cldn11 | 0.00296 | -1.65 |
| | Erbb3 | 0.00709 | -1.62 |
| | Tmem88b | 0.00151 | -1.60 |
| | Lpar1 | 0.00189 | -1.60 |
| | Bmp4 | 0.00236 | -1.56 |
| | Mbp | 0.00098 | -1.55 |
| | Fa2h | 0.00118 | -1.55 |
| | Pllp | 0.01043 | -1.47 |
| | Tspan2 | 0.00225 | -1.43 |
| | Plp1 | 0.00255 | -1.41 |
| | Sox10 | 0.00914 | -1.37 |
| | Tprn | 0.03378 | -1.35 |
| | Gamt | 0.00530 | -1.34 |
| | Gltp | 0.02723 | -1.33 |
| | Galnt6 | 0.03866 | -1.30 |
| | Dock5 | 0.00360 | -1.29 |
| | Gm98 | 0.02498 | -1.27 |
| | Prr18 | 0.02808 | -1.22 |
| | Cpm | 0.04832 | -1.20 |
| | Qdpr | 0.00424 | -1.20 |
| | Adamts4 | 0.00337 | -1.18 |
| | Efhd1 | 0.02822 | -1.10 |
| | Gsn | 0.00434 | -1.07 |
| | Arhgef10 | 0.00276 | -1.02 |
| | 9630013A20Rik | 0.01037 | -1.02 |
| | | | |
| | Tmem141 | 0.04560 | 1.04 |
| | Rbpjl | 0.03948 | 1.61 |

| | Gene | *p*-value | F.C.($log_2$) |
|---|---|---|---|
| Neurons | Lhx5 | 0.02313 | -1.27 |
| | Bmp5 | 0.04600 | -1.17 |
| | | | |
| | C030023E24Rik | 0.00924 | 1.07 |
| | Mia1 | 0.00903 | 1.43 |
| | Pla2g4e | 0.01884 | 1.83 |
| | Pth2r | 0.01704 | 2.14 |

| | Gene | *p*-value | F.C.($log_2$) |
|---|---|---|---|
| Astrocytes | Nmb | 0.01456 | -1.23 |
| | Scara3 | 0.00411 | -1.13 |
| | | | |
| | Mamdc2 | 0.00772 | 1.08 |
| | Sp6 | 0.04233 | 1.22 |
| | Exd1 | 0.02362 | 2.09 |

| | Gene | *p*-value | F.C.($log_2$) |
|---|---|---|---|
| Microglia | Cd80 | 0.01424 | -1.26 |
| | | | |
| | Tgfb1 | 0.04308 | 1.02 |
| | H2-Eb1 | 0.00344 | 1.05 |
| | Cst7 | 0.03294 | 1.08 |
| | Nfam1 | 0.00884 | 1.37 |
| | Il4ra | 0.04537 | 1.43 |
| | Clec4a1 | 0.04750 | 1.49 |
| | Card9 | 0.01122 | 1.55 |

| | Gene | *p*-value | F.C.($log_2$) |
|---|---|---|---|
| Endothelial cells | Rasip1 | 0.03193 | -1.62 |
| | | | |
| | Clec14a | 0.03186 | 1.06 |
| | Ifi47 | 0.01498 | 1.11 |
| | Hspa12b | 0.04922 | 1.11 |
| | Fam129a | 0.02479 | 1.57 |
| | Mmrn1 | 0.02199 | 1.95 |
| | Aplnr | 0.03804 | 2.00 |
| | Fam124b | 0.00127 | 2.32 |

Expanded list of significantly enriched genes as visualized in Figure 2. Blue text indicates down-regulated genes and red indicates up-regulated genes. F.C.; fold change. N=3 mice per group.

**Supplementary Table 3. Summary of qRT-PCR primers used for validation.**

| Target | Sequence | Product (bp) |
|---|---|---|
| mBubR1 #1-F | ACCAGGCCCTCATCATAAAG | 87 |
| mBubR1 #1-R | AAGAAGACCTGGAGAAGCCA | |
| mBubR1 #2-F | AGCTGAAAGAACGAAGGGAA | 93 |
| mBubR1 #2-R | TCAGCCTCCTCTCCATCTCT | |
| mOilg1-F | CCACCACAACTCACCCACTG | 65 |
| mOilg1-R | ACGGATACGAGAATAGCCCG | |
| mOlig2-F | CGCAGCGAGCACCTCAAATCTAA | 81 |
| mOlig2-R | CCCAGGGATGATCTAAGCTCTCGAA | |
| mMyrf-F | TGGCAACTTCACCTACCACA | 160 |
| mMyrf-R | GTGGAACCTCTGCAAAAAGC | |
| mSox10-F | AGCCCAGGTGAAGACAGAGA | 146 |
| mSox10-R | AGTCAAACTGGGGTCGTGAG | |
| mNkx2.2-F | CCTCCCCGAGTGGCAGAT | 74 |
| mNkx2.2-R | GAGTTCTATCCTCTCCAAAAGTTCAAA | |
| mCnp-F | GTTCTGAGACCCTCCGAAAA | 108 |
| mCnp-R | CCTTGGGTTCATCTCCAGAA | |
| mMal-F | TCACACTGGATGCAGCCTACC | 71 |
| mMal-R | CAGGGCTTCCAGAACTGAGG | |
| mMog-F | ATGAAGGAGGCTACACCTGC | 123 |
| mMog-R | CAAGTGCGATGAGAGTCAGC | |
| mMag-F | AACCAGTATGGCCAGAGAGC | 133 |
| mMag-R | GTTCCGGGTTGGATTTTACC | |
| mMbp-F | CCCGTGGAGCCGTGATC | 81 |
| mMbp-R | TCTTCAAACGAAAAGGGA | |
| mPlp-F | GTATAGGCAGTCTCTGCGCTGAT | 201 |
| mPlp-R | AAGTGGCAGCAATCATGAAGG | |
| mGapdh-F | ACCCAGAAGACTGTGGATGG | 171 |
| mGapdh-R | CACATTGGGGGTAGGAACAC | |
